# Silencing of the sulfur rich α-gliadin storage protein family in wheat grains (*Triticum aestivum* L.) causes no unintended side-effects on other metabolites

**DOI:** 10.3389/fpls.2013.00369

**Published:** 2013-09-17

**Authors:** Christian Zörb, Dirk Becker, Mario Hasler, Karl H. Mühling, Victoria Gödde, Karsten Niehaus, Christoph-Martin Geilfus

**Affiliations:** ^1^Institute of Biology, University LeipzigLeipzig, Germany; ^2^Biocentre Klein Flottbek, EBBT, University of HamburgHamburg, Germany; ^3^Lehrfach Variationsstatistik, Christian Albrechts University of KielKiel, Germany; ^4^Institute of Plant Nutrition and Soil Science, Christian Albrechts University KielKiel, Germany; ^5^Department of Proteome and Metabolome Research, Faculty of Biology, Bielefeld UniversityBielefeld, Germany

**Keywords:** sulfur, wheat, gliadin, metabolites, Celiac disease, GC-MS

## Abstract

Wheat is an important source of proteins and metabolites for human and animal nutrition. To assess the nutritional quality of wheat products, various protein and diverse metabolites have to be evaluated. The grain storage protein family of the α-gliadins are suggested to be the primary initiator of the inflammatory response to gluten in Celiac disease patients. With the technique of RNA*i*, the α-gliadin storage protein fraction in wheat grains was recently knocked down. From a patient's perspective, this is a desired approach, however, this study aims to evaluate whether such a down-regulation of these problematic α-gliadins also has unintended side-effects on other plant metabolites. Such uncontrolled and unknown arbitrary effects on any metabolite in plants designated for food production would surely represent an avoidable risk for the consumer. In general, α-gliadins are rich in sulfur, making their synthesis and content depended of the sulfur supply. For this reason, the influence of the application of increasing sulfur amounts on the metabolome of α-gliadin-deficient wheat was additionally investigated because it might be possible that e.g., considerable high/low amounts of S might increase or even induce such unintended effects that are not observable under moderate S nutrition. By silencing the α-gliadin genes, a recently developed wheat line that lacks the set of 75 corresponding α-gliadin proteins has become available. The plants were subsequently tested for RNA*i-induced* effects on metabolites that were not directly attributable to the specific effects of the RNA*i*-approach on the α-gliadin proteins. For this, GC-MS-based metabolite profiles were recorded. A comparison of wild type with gliadin-deficient plants cultivated in pot experiments revealed no differences in all 109 analyzed metabolites, regardless of the S-nutritional status. No unintended effects attributable to the RNA*i*-based specific genetic deletion of a storage protein fraction were observed.

## Introduction

Wheat is an important source of proteins and metabolites for human and animal nutrition and is counted among the “big three” cereal crops, with over 600 million tonnes being harvested annually (Shewry, 2009). To assess the nutritional quality of wheat products, different protein families such as storage proteins and diverse metabolites need to be evaluated. Wheat storage proteins are some of the most important proteins for human consumption and, *via* baking, contribute to our daily bread. Gliadins have a Janus-faced nature for Man because, on the one hand, they contribute to baking quality by building sulfur bridges and modulating rheological processes in bread making (Wieser, 1995). On the other hand, they are sources for allergic reactions in the human intestine and might cause Celiac diseases. One fraction of the wheat storage proteins, the sulfur-rich α-gliadins, is known to initiate Celiac disease, a life-long gluten-sensitive autoimmune disease of the small intestine (Shan et al., 2002). Celiac disease represents one of the most common genetic diseases with its prevalence being estimated to be approximate 0.5–3% in various parts of the world (Shewry, 2009; Gujral et al., 2012). The affected people have no other choice than to avoid gliadins dependent on their grade of allergic reaction or rigorously to abandon gluten-based products. A reduction of gliadins in flour-based products, such as bread or noodles, might contribute to better tolerance by those consumers who have to avoid large amounts of gliadins. An α-gliadin-free transgenic wheat line might represent a strategy in the dietary minimization of allergens. However, especially in some parts of Europe, public and scientific concerns have been raised about the environmental and food safety of genetically modified crops (Nap et al., 2003; Johnson et al., 2007). Moreover, serious concerns have been raised that these transgenic approaches will have unintended side-effects on, for example, single metabolites or the entire metabolome in the plant (McHughen, 2012), e.g., due to a tailback of metabolites that in turn may shift equilibrium reactions to one or the other side.

Recently, a wheat line has become available that lacks the α-gliadin proteins in the grains. This was achieved by simultaneously silencing the set of 75 corresponding α-gliadin genes using the technique of RNA*interference* (RNA*i*) (Becker et al., 2012). As measured by RP-HPLC and two dimensional proteome analysis (Becker et al., 2012), the α-gliadin family has been proven to be completely eliminated in the transgenic wheat. It was one the aim of this study to elucidate whether the RNA*i* approach has unintended side-effects on other metabolites that were not directly attributable to the specific effects of the RNA*i*-approach on the α-gliadin proteins. Since α-gliadins are sulfur-rich, we have furthermore cultivated these α-gliadin-deficient plants with increasing amounts of sulfur in pot experiments, because it might be possible that e.g., considerable high/low amounts of S might increase or even induce such unintended effects that are not observable under moderate S nutrition. For example, high S-supply inevitably increases S-containing amino acids such as cysteine and methionine (Scherer, 2001; Granvogl et al., 2007; Rennenberg et al., 2007; Howarth et al., 2008) and may therefore increase the amounts of α-gliadins that contain these S-rich amino acids and therefore contain large numbers of disulfide bonds. However, in the α-gliadin deficient wheat line that finally lacks the messenger RNA that codes for the α-gliadin, these S-rich amino acids might accumulate without being incorporated into α-gliadins leading to the incorporation of those amino acids into other metabolites. This may shift equilibrium reactions causing unintended and uncontrolled side-effects on other metabolites that are not desired in the perspective of food security.

The aim of our study has been to investigate whether the RNA*i*-approach affects other metabolites than the targeted α-gliadin under conditions of varying S-supply. For this 109 detectable metabolites were screened by means of GC-MS analysis. Ripe plants cultivated in pot experiments were divided into (i) whole grains, (ii) husk plus rachis, and (iii) the remaining straw whereas the wild type was compared against the transgenice line. The gas chromatography-mass spectrometry-based metabolite profiles were compared by calculating a principal component followed by a more individual multiple contrast tests.

## Materials and methods

### Plant material and growth conditions

For the development of the α-gliadin knock down plants, the technique of RNA*i* was applied to silence more than 75 α-gliadin genes as described in Becker et al. (2012). These genetically modified winter wheat (*Triticum aestivum* L.) plants were tested and used in this study together with the corresponding wild type variety cv. Florida. The α-gliadin RNA*i* knock-down plants and the wild type were grown in parallel under defined conditions in a greenhouse (Zörb et al., 2012). Pots were filled with identical sulfate-depleted soil in Mitscherlich pots containing 6 kg soil (1/3 loam and 2/3 quartz-sand) and increasing amounts of MgSO_4_. Loam (type Klein-Linden, pH 5.1) had an SO_4_-sulfate concentration of 10.2 mg kg^−1^, and glowed quartz-sand (0.6–1.2 mm 7FG, Dorsilit, Gebrüder Dorfner GmbH&Co., Hirschau, Germany) was free of sulfur. For watering, only de-ionized water was used to avoid any entry of further nutrients. After the sowing of 24 kernels, 16 uniform plants were cultivated per pot. The experiment was conducted during 2008–09 with an average day/night temperature of 18/26°C (summer) and 8/15°C (winter) and a photoperiod of 14 h (~400 μmol m^−2^ s^−1^ PAR) for 9 months at a RH of ~70 ± 10%. Three different sulfur (S) fertilization rates were applied by adding 0 (none), 0.1 (moderate), and 0.2 g (high) S/pot before sowing (EC 01). Each treatment was biologically replicated five times. Nitrogen was applied at a single rate (1.0 g N, NH_4_NO_3_) at three different times [EC 01, sowing; EC 11, stocking; EC 43, ear development, developmental stages according to Lancashire et al. (1991)] during the growing season. Phosphorus (5.0 g CaHPO_4_/pot), K (3.4 g KCl/pot), Mg (1.0 g MgCO_3_/pot), and the minor elements were applied at recommended rates for optimal growth. No nutrient deficiency of plants, except for that of sulfur under non-fertilization, was visible.

### Harvest of plant material

At EC 92, ripe plants were harvested and fractionated into (i) whole grains (milled to whole grain flour), (ii) husk plus rachis (blended due to threshing), and (iii) the remaining straw (all leaves and stems). Straw, husk plus rachis were ground to a fine homogeneous powder by using a ball mill equipped with a 0.5 mm sieve (Retsch, Haan, Germany). The whole grain flour had a water content of 13% and was stored at −20°C prior analysis. Husk, rachis, and straw have been dried to constant weight at 50°C.

### Metabolite extraction

The metabolites were extracted from 10 mg material with 1 mL 80% methanol, containing 10 μM ribitol as an internal standard, in a Precellys24 Instrument (Peqlab, Erlangen, Germany), by using 1 mm zirconia beads (Roth, Karlsruhe, Germany). Extracts were treated three times at 6.5 m/s for 45 s. After 20 min centrifugation at 15,000 g at room temperature, the clear supernatant was transferred to 1 mL glass vials (Supelco, Bellfonte, California) and evaporated in a nitrogen stream. Metabolites were derivatized as described elsewhere (Zörb et al., 2013).

### GC-MS analysis

Sample volumes of 1 μL were analyzed with a Trace GC gas chromatograph coupled to a PolarisQ ion trap mass spectrometer equipped with an AS2000 auto sampler (Thermo Electron, Dreieich, Germany). Derivatized metabolites were evaporated at 250°C in splitless mode and separated on a 30 m × 0.25 mm RTX-5MS capillary column with 0.25 μm coating equipped with an integrated 10 m guard column (Restek, Bad Homburg, Germany). Helium carrier gas flow was adjusted to 1 mL/min. The interface temperature was set to 250°C and the ion source temperature to 220°C. Oven temperature was kept constant for 3 min at 80°C and subsequently raised to 325°C at 5°C/min. The system was equilibrated for 5 min at 80°C after each analysis. Mass spectra were recorded at 1 scan/s with a scanning range of 50–750 m/z. In total, 109 metabolites were identified (Supplemental Material 1) by comparison with purified standards and by using the NIST 2005 database (NIST, Gaithersburg, Md.). In addition, the freely available Golm Metabolome Database (Kopka et al., 2005) was of particular help for the identification of several metabolites. Relative levels (Supplemental Material 2) of selected metabolites were determined automatically by integrating the peak areas of selective ions (Fiehn et al., 2000) and the processing setup implemented in Xcalibur 1.4 software (Thermo Electron, Dreieich, Germany). Relative response ratios were calculated by normalizing the respective peak areas to the peak area of the internal standard and dividing the value by the weight of the extracted sample. Concerning reproducibility, five aliquots of one sample were extracted in parallel and taken separately through the sample preparation and GC-MS analysis procedure for all individual metabolites. From these five analyses, standard deviations were calculated as described elsewhere (Zörb et al., 2013). In order to determine the accuracy of the system, seven internal technical replications were made; values were only accepted when the standard error was below 5%. In addition, calibration curves for 28 commercially available metabolites were generated. These measurements revealed the linearity of the detection for most metabolites in the range of 100 fmol to 1 nmol. Samples were randomized prior to GC-MS injection in order to prevent bias attributable to instrument performance.

### Statistics for GC-MS analysis

In total, 109 metabolites were detected in each of the factor groups. Factor groups were the organs (grain, straw, husk blended with rachis) and the sulfur fertilization rates (0 g, 0.1 g, or 0.2 g S/pot). In order to test for differences in the metabolome between all the comparative groups, hundreds of comparisons were necessary. However, the usual tests for mean differences were not developed for such large data sets and were therefore not optimal, because of the large sample size associated with the risk of false-positive or false-negative test decisions. For this reason, a principal component analysis in combination with multiple contrast tests for ratios of means including multiplicity adjustment for the single metabolites were chosen to provide a robust and careful statistical evaluation of the data. Thus, only the most robust and significant physiological effects were considered in this work.

#### Principal component analysis

The statistical software R (2012) was used to evaluate the data. The statistical evaluation started with a principal component analysis according to Hartung and Elpelt (1999). Supplemental Material 3 shows the resulting screeplot of the PCA. Since the first principal component represents, at 30.2%, the most likely weighting of the principal components, the subsequent multiple contrast tests for ratios of means were based on this first principal component. The following analysis consisted of two steps. First, the data were transformed into a new variable based on the first principal component, because an analysis of this new variable allowed conclusions about all metabolites together. After this, multiple contrast tests for ratios of means according to Hasler and Hothorn (2008) were conducted. The means related to the influence factor organs (straw, grain, husk plus rachis) were compared for each level of the remaining influence factor sulfur fertilization rates (0; 0.1; 0. 2 g/S per pot). This was carried out based on a corresponding cell means model (Schaarschmidt and Vaas, 2009). In a further step, the 28 most relevant metabolites were selected for a further, more individual analysis. These metabolites are statistically most important as their corresponding loadings of the principal component analysis had the highest absolute values. The same multiple contrast tests as in the first step were conducted but simultaneously for the 28 metabolites. An additional multiplicity adjustment according to Holm (1979) for the number of metabolites, ignoring their correlations, was carried out.

## Results

### Genotypic differences in the metabolite profiles

**Figure 1:**
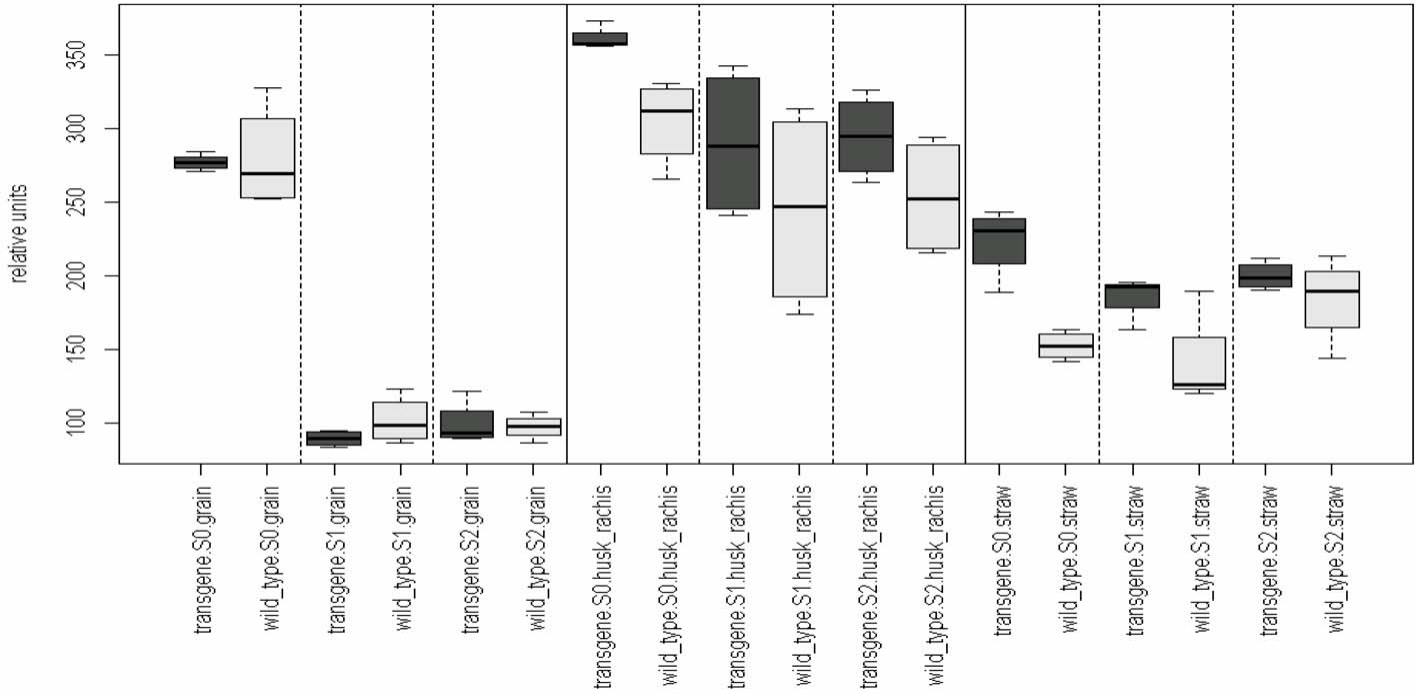
**Genotypic effect of the RNAi-approach on the metabolome.** Comparison of the means of the influence factor “genotype” (transgene vs. the wild type) for each level of the remaining influence (organs and sulfur fertilization rate). Multiple contrast tests for ratios of means. None of the comparisons revealed significant mean differences between the means. For example, in the first comparison, the mean of group “transgene, S0, grain” equals the mean of the group “wild type, S0, grain, S0.” The remaining comparisons can be interpreted in a similar way. Comparisons are indicated by the framed dotted lines. No mean differences exist at a *p*-value < 0.05. Light-gray, wild-type control; dark-gray, transgene wheat line. Wild type, cv. Florida; transgene, alpha-gliadin knock down. Sulfur treatment: S0, 0 g S/pot; S1, 0.1 g S/pot; S2, 0.2 g S/pot.

In order to analyze whether the silencing of gliadins has unintended effects on the metabolomic composition of several wheat organs, a GC-MS-based metabolite profiling approach was chosen. A comparison of the metabolome of both genotypes (transgene wheat line *vs*. the wild type control) for each level of the remaining influence (organs: grain, straw, husk blended with rachis; and sulfur fertilization: S1, S2, S3) revealed no genotypic differences in the metabolite pattern between the transgenic wheat line and the wild type control (Table 1). All comparative groups between the transgenic line and the control matched in terms of their metabolite profiles (Figure 1).

**Table 1:** **Testing for genotypic differences in the metabolome**.

**Comparison**	**Organ(s)**	**S-fertilization [g S/pot]**	**Ratio**	***p*.value.adj**	**Test decision**
Transgene vs. Wild type	Grain	0	0.990	1	No differences between metabolites
		0.1	0.880	0.7536	No differences between metabolites
		0.2	1.021	1	No differences between metabolites
Transgene vs. Wild type	Husk and rachis	0	1.188	0.1515	No differences between metabolites
		0.1	1.182	0.9312	No differences between metabolites
		0.2	1.162	0.6434	No differences between metabolites
Transgene vs. Wild type	Straw	0	1.466	0.0543	No differences between metabolites
		0.1	1.307	0.3968	No differences between metabolites
		0.2	1.086	0.9175	No differences between metabolites

*Genotypic effect of the RNAi-approach on the metabolome. Comparison of the means of the genotypes (transgene vs. the wild type) for each level of the remaining influence (organs and S-fertilization rate). Multiple contrast tests for ratios of means. Column “Ratio” refers to the ratio of the two means of the corresponding comparison. For example, in row 1, the mean of group “grain, S0, transgene” is 0.990 times the mean of group “grain, S0, wild type.” These two means are not significantly different (p = 1). The remaining comparisons can be interpreted in a similar way. Thus, no metabolome differences can be seen between the two genotypes, either in the three different organs or as caused by the variable sulfur supply. Wild type, cv. Florida; transgene, alpha-gliadin knock down. Sulfur treatment: S0, 0 g S/pot; S1, 0.1 g S/pot; S2, 0.2 g S/pot*.

### Influence of a variable sulfur fertilization rate on the metabolite profile

**Figure 2:**
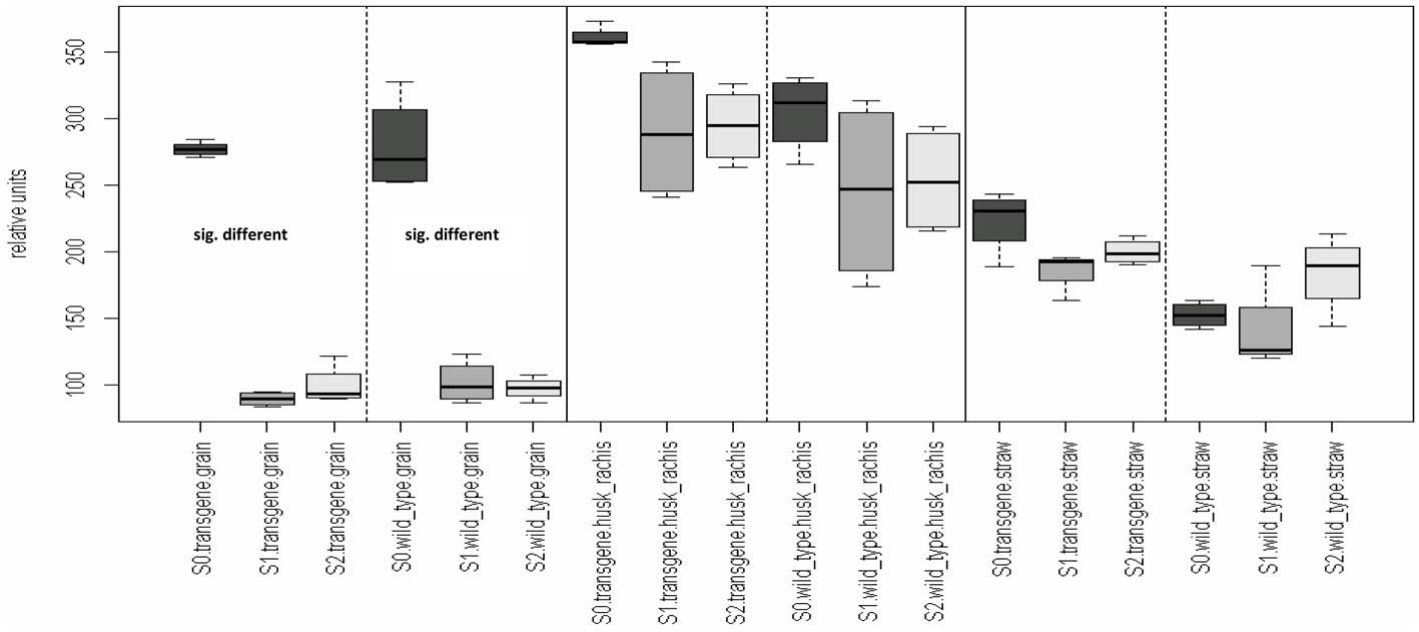
**Organ-specific effects of varying sulfur fertilization rates on the metabolome.** Comparison of the means of the influence factor “S-fertilization rate” (S0, S1, S2) for each level of the remaining influence (genotpye and organs). Multiple contrast tests for ratios of means. Comparisons are indicated by the framed dotted lines. In the first comparison, the mean of group “S0, transgene, grain” differs from the mean of the two groups “S1, transgene, grain” and “S2, transgene, grain.” This difference also occurred in the grains of the wild type. No other significant mean differences in any of the other comparisons at *p*-value < 0.05 were detected. Light-gray, S2; moderate gray S1; dark-gray, S0. Wild type, cv. Florida; transgene, alpha-gliadin knock down. Sulfur treatment: S0, 0 g S/pot; S1, 0.1 g S/pot; S2, 0.2 g S/pot.

A variable sulfur supply influenced the quantitative composition of the grain metabolome in both the wild type control and the transgenic wheat line (Table 2A). This effect was only observed in the grains and not in the straw nor in the “husk plus rachis”-fraction. All plants that were grown with S1 and S2 showed higher grain metabolite concentration than plants grown at S0 (Figure 2). In contrast to the grains, the metabolite concentration in the fraction “husk plus rachis” and in the straw was not affected by the variable sulfur supply.

**Table 2A:** **Testing the influence of variable sulfur fertilization rates on the metabolite profile i.e., metabolome**.

**Comparison**	**Genotype**	**Organ(s)**	**Ratio**	***p*-value.adj**	**Test decision**
S1 vs. S0	Wild type	Grain	0.363	0.006	Significant differences between metabolites
S2 vs. S0			0.348	0.013	Significant differences between metabolites
S2 vs. S1			0.958	0.999	No differences between metabolites
S1 vs. S0	Transgene	Grain	0.323	0.00	Significant differences between metabolites
S2 vs. S0			0.359	0.001	Significant differences between metabolites
S2 vs. S1			1.111	0.892	No differences between metabolites
S1 vs. S0	Wild type	Husk and rachis	0.804	0.772	No differences between metabolites
S2 vs. S0			0.831	0.546	No differences between metabolites
S2 vs. S1			1.034	1	No differences between metabolites
S1 vs. S0	Transgene	Husk and rachis	0.8	0.360	No differences between metabolites
S2 vs. S0			0.813	0.122	No differences between metabolites
S2 vs. S1			1.017	1	No differences between metabolites
S1 vs. S0	Wild type	Straw	0.923	0.996	No differences between metabolites
S2 vs. S0			1.208	0.574	No differences between metabolites
S2 vs. S1			1.310	0.58	No differences between metabolites
S1 vs. S0	Transgene	Straw	0.822	0.381	No differences between metabolites
S2 vs. S0			0.895	0.666	No differences between metabolites
S2 vs. S1			1.080	0.825	No differences between metabolites

*Organ-specific effects of variable S-fertilization rates on the whole metabolome. Comparison of the means of the S-fertilization rates (S0, S1, S2) for each level of the remaining influence factors (genotpye and organs). Multiple contrast tests for ratios of means. Column “Ratio” refers to the ratio of the two means of the corresponding comparison. For example, in row 1, the mean of group “grain, wild type, S1” is 0.363 times the means of group “grain, wild type, S0.” These two means are significantly different (p = 0.0064). In other words, fewer metabolites are present in the grains of the wild type that was not supplied with S compared with those that received treatment with 0.1 g S/pot. The remaining comparisons can be interpreted in a similar way. Significant differences of the mean (p = 0.05) occurred only in the grains under increasing sulfur supply conditions. Wild type, cv. Florida; transgene, alpha-gliadin knock down. Sulfur treatment: S0, 0 g S/pot; S1, 0.1 g S/pot; S2, 0.2 g S/pot*.

### Influence of a variable sulfur fertilization rate on single metabolites

To test which of the metabolites were most responsible for the metabolite profile differences between the variable sulfur fertilization rates in the grains of both genotype (as demonstrated in Table 1 and Figure 1), a multiple contrast test with an additional multiplicity adjustment was performed. Lying beneath other metabolites involved in the PCA (Supplemental Material 4), β-amino isobutyric acid was found to be the highly responsible for the sulfur-related differences in the grains of the wild type, because its concentration in the grains increased significantly with increasing sulfur rates (Table 2B). When α-gliadins were silenced, the amino acids alanine, glycine, serine, homoserine, and tyrosine proved to be highly responsible for sulfur-related differences in the grains of the knock-down genotype. The concentration of these metabolites increased with increasing sulfur supply (Table 2B).

**Table 2B:** **Testing the influence of variable sulfur fertilization rates on single metabolites**.

**Summary of comparison from table 2A**			**Multiplicity adjustment for the single metabolites according to Holm**			
**Genotype**	**Comparison**	**Mean difference**	**Metabolite**	**Ratio**	***p*-value**	**Test decision**
Wild type	S1 vs. S0	Yes	β-aminoisobutyric acid	0.147	0.0151	Significantly less in the S0 treatment
	S2 vs. S0	Yes	β-aminoisobutyric acid	0.171	0:0015	Significantly less in the S0 treatment
	S2 vs. S1	No	–	–	–	–
Transgene	S1 vs. S0	Yes	Alanine	0.158	0.000	Significantly less in the S0 treatment
			Glycine	0.131	0.054	Significantly less in the S0 treatment
			Serine	0.108	0.026	Significantly less in the S0 treatment
			Homoserine	0.228	0.062	Significantly less in the S0 treatment
			Tyrosine	0.255	0.063	Significantly less in the S0 treatment
			Threonine	0.100	0.042	Significantly less in the S0 treatment
	S2 vs. S0	Yes	Alanine	0.158	0.000	Significantly less in the S0 treatment
			Glycine	0.147	0.003	Significantly less in the S0 treatment
			Serine	0.116	0.000	Significantly less in the S0 treatment
			Homoserine	0.206	0.045	Significantly less in the S0 treatment
			Tyrosine	0.236	0.049	Significantly less in the S0 treatment
	S2 vs. S1	No	–	–	–	–

*Testing the influence of variable sulfur fertilization rates (S0, S1, S2) on the single metabolites. Multiple contrast tests for 28 metabolites with an additional multiplicity adjustment. Columns “Summary of comparison from Table 2A” summarize the core results from the multiple contrast tests for the grains fully presented in Table 2A. The other columns show which of the 28 metabolites contribute most to the significant mean differences described in Table 2A. Only comparisons with a p-value < 0.1 are shown for the sake of brevity. As an example, the mean of group “wild type, S1” is 0.147 times the means of group “wild type, S0.” These two means are significantly different (p = 0.0151). In other words, less β-amino-isobutyric acid is present in the grains of the S0 treatment of wild type plants compared with the grains of the wild type plants receiving the S1 treatment. The remaining comparisons can be interpreted in a similar way. Wild type, cv. Florida; transgene, alpha-gliadin knock down. Sulfur treatment: S0, 0 g S/pot; S1, 0.1 g S/pot; S2, 0.2 g S/pot*.

## Discussion

Deletion of a whole family of the storage protein fraction might be assumed to cause secondary effects on the whole plant metabolism and in particular on the seed metabolome (McHughen, 2012). Possible unintended effects of α-gliadin silencing may than influence on the quality of grain based food. In order to test for such unintended effects on metabolites caused by an RNA*i* approach, i.e., the deletion of 75 α-gliadins in wheat grains, GC-MS-based metabolite profiles were recorded. These profiles were used for a subsequent statistical analysis performed to determine genotypic differences between the transgenic wheat line and the wild type control. The deletion of a storage protein that represents a major sink for carbon-, nitrogen-, and sulfur-containing metabolites might realistically be considered to disturb the regular sink-source relationships or the internal translocation of metabolites within the wheat plant. Surprisingly, the metabolome of the transgenic plants totally matched and equaled the metabolome of the corresponding wild type plants. No significant differences in metabolites could be detected between the two genotypes, either in the three different organs (straw, husk plus rachis, grains) or under the variable S-supply conditions (Table 1 and Figure 1). This indicates that the substantial genetic modification to silence 75 α-gliadin genes did not arbitrarily affect metabolites in any of the analyzed wheat organs. Becker et al. (2012) have been able to demonstrate, by means of two-dimensional proteome analysis that the lack of the α-gliadins is specifically compensated by an increase of albumins/globulins, ω-gliadins, γ-gliadins, and HMW glutenin subunits. It is possible that unintended metabolic flux-disturbances do not occur because other storage proteins replace the α-gliadins plastically as physiological C-, N-, and S-sinks.

In contrast, an increase in the general sulfur supply of plants resulted in significant changes in the metabolome. However, this was observed in conformity for both genotypes especially by a comparison of 0.1 and 0.2 *vs*. cero S fertilization (Table 2A and Figure 2) and, by means of this, can not represent a genotypic difference caused by the RNA*i* approach. This shows that the effect of alterations in the availability of a single mineral nutrient on the metabolite composition is greater than that caused by RNA*i*-based genetic deletion of a storage protein fraction. When more sulfur was added as fertilizer, quantitatively more metabolites were synthesized in the grains. When α-gliadins were silenced, increases in the concentration of the amino acids alanine, glycine, serine, homoserine, and tyrosine were most responsible for the sulfur-induced increase in the grain metabolome (Table 2B). In the wild type control, an increase of β-aminoisobutyric acid was the most responsible for the sulfur-related increase in the grain metabolome (Table 2B). Since the amino acids alanine, glycine, serine, homoserine, and tyrosine are precursors for the α-gliadins, an accumulation of those amino acids under increasing sulfur supply might be explained by a tailback attributable to the lack of α-gliadin, representing a sink of those amino acids. Such an effect seems to be directly related to the aim of the silencing process and can not be interpreted as a unintended side effects. We could not find that these accumulated metabolites arbitrarily influence on other pathways as, otherwise, multiple contrast test for the single metabolites would have indicated this.

## Conclusion

This study is the first to assess the differences of the metabolome after silencing a whole storage protein family of wheat grain. A GC-MS-based principal component analysis that compared the metabolome of wild type with the metabolome of the α-gliadin-deficient wheat revealed no differences in all 109 analyzed metabolites, regardless of the S-nutritional status or the organ (grain, husk together with rachis or straw). No unintended effects attributable to the RNA*i*-based specific genetic deletion of a storage protein fraction on other metabolites could be demonstrated. Remarkably, the effect of the availability of a single mineral nutrient on the metabolome was much higher compared to that of the RNA-silencing.

### Conflict of interest statement

The authors declare that the research was conducted in the absence of any commercial or financial relationships that could be construed as a potential conflict of interest.

## Abbreviations

PCA: principal component analysis
GC-MS: gas chromatography–mass spectrometry
RNAi: RNA interference.
