# Supplementary material for: Silencing of the sulfur rich α-gliadin storage protein family in wheat grains (Triticum aestivum L.) causes no unintended side-effects on other metabolites
Source: Front Plant Sci. 2013 Sep 17;4:369. doi: 10.3389/fpls.2013.00369 (PMC3775129; doi:10.3389/fpls.2013.00369)
Supplement: Supplementary file 1 [file DataSheet1.DOC]

**Supplemental Material 1**

Loadings for the first four principal components (PC) of the PCA for each of the metabolites. The higher the absolute value of a certain loading, the higher the potential contribution of the corresponding metabolite to the mean differences (due to the organs, genotypes and S-fertilization rate).

**Metabolite** **PC1 PC2 PC3 PC4**

Pyruvate_174 0.052137 -0.128545 0.031528 -0.147169

Alanine_116 0.131482 -0.082657 0.121902 0.037743

β-Aminoisobutyric acid _130 0.008300 -0.160135 0.135488 -0.084000

Valine_144 0.138995 -0.098128 0.034957 -0.103359

α-Ketocaproat_110 0.114110 -0.076418 0.020503 -0.046346

Urea_189 0.023801 -0.098949 0.223383 -0.006327

Ethanolamine_174 0.165519 -0.002500 0.045634 0.021231

Leucine_158 0.062171 -0.147569 0.070913 -0.154878

Isoleucine_158 0.121827 -0.111506 0.010734 -0.127664

Proline-N-Odi_tms_142 0.152278 -0.036814 -0.095598 -0.058790

Glycine_174 0.047216 -0.154054 0.143295 -0.061681

Succinate_247_409 0.135167 0.054843 0.073984 0.087242

Glycerate_189_292 0.119537 0.134905 0.058803 -0.004777

Uracil_255_241 0.153946 -0.012759 -0.095749 0.043508

Fumarate_245 0.028032 -0.045931 0.226450 0.065972

Serine_204 0.128602 -0.085668 0.117824 0.051550

Threonine_101 0.072352 -0.135497 0.146966 -0.080245

Thymine_255 0.152188 -0.012891 -0.048476 0.123684

S-Methylcysteine_218 0.090175 -0.003089 0.014347 0.043231

β-Alanine_248 0.159639 -0.033417 0.032196 0.109041

Orcinole_253_268 0.067806 0.020485 0.000282 0.096296

Homoserine_3TMS_218 0.102892 -0.025910 -0.133935 -0.120292

Glutamine_3TMS_155 0.133909 -0.027365 0.033120 0.116056

Malate_245_307 0.142241 0.078248 0.037753 0.047460

Methionine_176 0.013877 -0.144686 -0.002064 -0.191039

Cytosine_2TMS_240_257_ 0.114507 -0.015616 -0.019934 -0.028050

L-Aspartate_3TMS_100_188_232 0.030182 -0.026222 0.193690 0.144713

Cinnamic acid_131_161_205 0.043223 -0.041122 0.139354 -0.044872

Cysteine_220 0.095134 0.131864 0.078612 0.007018

II-Hydroxyglutarate_203_247 0.149102 -0.006400 -0.118098 0.006047

α-Ketoglutarate_198 0.002757 -0.160001 0.064273 -0.146174

Asparagine_4TMS_MEOX1_216 0.090252 -0.079940 0.149171 0.151561

Glutamate_230_246 0.041511 -0.056242 0.212631 0.083749

Phenylalanine_2TMS_192 0.117962 0.070089 -0.033971 -0.154590

Asparagine_4TMS_MEOX2_216 0.082101 -0.097539 0.163153 0.124766

Xylose-Me_OX1_5TMS_217_307 0.019558 0.138428 0.054317 -0.157662

Homocysteine_128_234 0.157716 0.000204 -0.053459 -0.017274

Asparagine_3TMS_231 0.088770 -0.084088 0.153980 0.152410

Ribose_217 -0.013599 0.163297 0.066112 -0.151784

II-Aminoadipat_260 0.162001 -0.010202 -0.044478 0.072929

Rhamnose_MeOX_4TMS_277 0.062889 0.033235 -0.110821 -0.068910

cis-Aconitat_229 0.155124 0.014662 -0.052856 0.094810

Dihydroacetonphosphate_ MEOX1_400 -0.048163 -0.066510 0.130190 0.003977

Glycerol-3-P_357 -0.024900 -0.147444 -0.150882 -0.013769

N-Acetyl-glucosamine_156_274 0.083900 -0.125101 0.140414 -0.051698

Glycerate-2-P_299_315_459 0.045066 0.107484 0.031262 -0.163010

Syringaldehyde_253_283 0.083721 0.150734 0.039846 -0.078963

1-Methyl-L-histidine_196 0.071128 -0.046339 0.096733 0.089409

Shikimate_204 0.146011 -0.023233 -0.017657 -0.140537

Citrullin-Ornithine-Arginine_4TMS_142 0.109384 -0.104542 -0.070701 -0.108223

Glycerate-3-P_227_299_459 0.085590 -0.048052 -0.032583 -0.052761

Arginine-NH3_3TMS_157_256 0.070853 -0.137370 -0.004592 -0.184058

Citrate_257 0.158935 0.034738 -0.013504 0.086430

Isocitrate_245_319 0.152618 0.008058 -0.094877 0.089140

Pinitole_260 0.064105 -0.092576 0.116586 0.093872

II-Methylcitrate_MEOX1_287 0.162207 -0.009827 -0.052842 0.074076

II_Methylcitrate_MEOX2_287 0.156365 -0.027447 -0.079076 0.074110

Adenine_264 0.152046 -0.001851 -0.125607 -0.002858

Fructose_MEOX1_307 0.090605 0.150125 0.080651 -0.040608

Gluconate-1-5-lacton_129_220 0.043653 0.129075 0.085949 -0.073339

Fructose_MEOX2_307 0.086853 0.151324 0.081180 -0.043831

Galactose_MeOX1_5TMS_319 0.009568 0.147528 0.076867 -0.201444

Glucose_MEOX1_5TMS_319 0.064140 0.154964 0.056109 -0.135461

Lysine_156 0.109634 -0.128706 -0.010620 -0.117252

Histidine_154 0.113613 -0.127633 0.010076 -0.111211

Galactose_MeOX2_5TMS_319 0.014673 0.156857 0.065773 -0.178341

Erythrose-4-P_357 0.086376 0.092235 0.120981 0.086921

Glucose_MEOX2_5TMS_319 0.072817 0.154320 0.039411 -0.132766

Cinnamic acid_219_249_293_308 0.132270 0.113704 -0.038453 -0.015166

Coniferylaldehyde_MEOX1_218_248_278 0.070067 -0.062849 0.029395 -0.006637

Tyrosine_218 0.113483 -0.138386 -0.038536 -0.075110

Coniferylaldehyde_MEOX2_218_248_278 0.066162 0.009573 0.046738 0.026743

Mannitole_6TMS_217_319 0.009859 0.123693 0.172795 0.013495

Dihydro-Caffeic acid_179_398 0.112872 -0.083184 -0.044479 -0.043635

Glucuronic acid_MEOX1_333 -0.080600 0.026363 0.052555 -0.171304

trans-Chalcone_MEOX1_206_236 -0.026858 -0.020744 0.075084 0.077900

Glucuronic acid_MEOX2_333 0.016779 0.043590 0.120932 0.021763

Pantothenic acid_201 -0.054676 -0.144619 0.008707 0.049606

trans_Chalcone_MEOX2_206_236 0.046112 0.095801 0.080045 -0.002151

Gluconate_333 0.120931 0.060276 0.003391 -0.119936

III-Hydroxy-4-Methoxy_Cinnamic acid 0.084632 0.116908 0.127410 0.009505

Ferulic acid_308_323_338 0.093459 0.116275 0.144414 -0.044117

Sinapaldehyde_174_248 -0.021167 0.066658 0.002716 -0.201073

myo-Inositol_305 0.117342 0.117403 -0.000331 -0.023925

Ribose-5-P_315_299 0.078735 -0.015724 0.038267 0.192347

Ribulose-5-P_357 -0.024898 -0.093653 0.133708 0.024748

Spermidine_201 -0.072086 -0.155681 0.030293 -0.064298

L-Cystathionine_4TMS_128_218_245 0.104974 0.027391 0.007079 0.086110

Tryptophan_202 0.001780 -0.084895 -0.103000 0.073246

Sinapinic acid_323_338_368 0.121340 0.062943 0.061496 -0.037016

Cystine-218_266 0.020207 0.000098 -0.116370 -0.000613

Glucose-6-P_MEOX1_387 0.133270 0.029247 -0.151393 -0.057959

Myo-Inositol-P_318 0.097208 -0.124073 -0.130120 - 0.041123

Supplemental Material 1 (*continued*)

Gluconate-6-P_7TMS_333_387 0.105799 0.028057 -0.030624 0.017530

Adenosine_4TMS_236 0.147160 -0.055792 -0.111782 -0.019490

Saccharose_8TMS_361 0.097511 -0.119024 -0.042089 -0.130792

Cellobiose_361 0.067015 0.146348 0.109474 -0.023084

Maltose_480 -0.052514 -0.163428 0.041949 -0.113360

Trehalose_361 -0.035045 -0.042211 0.197372 -0.047511

Naringenin_545 0.074002 0.024746 -0.075798 -0.083514

Epicatechin_355_368 0.040737 0.119985 0.050397 -0.093528

Melibiose_MeOX1_8TMS_204_361 -0.014460 -0.105919 0.069182 -0.161884

Daidzein_383_398 -0.012338 0.110218 0.080137 -0.039661

Melibiose_MeOX1_8TMS_204_361 -0.014460 -0.105919 0.069182 -0.161884

Coumesterole_412 0.015992 0.012095 -0.137391 -0.016025

Apigenin_471 0.097577 -0.076730 -0.083892 0.048059

Quercetin_647 -0.021818 -0.068099 0.053517 -0.074187

Luteolin_559 -0.086289 -0.147567 0.039098 -0.075450

Supplemental Material 1 (*continued*)
