# Supplementary material for: Silencing of the sulfur rich α-gliadin storage protein family in wheat grains (Triticum aestivum L.) causes no unintended side-effects on other metabolites
Source: Front Plant Sci. 2013 Sep 17;4:369. doi: 10.3389/fpls.2013.00369 (PMC3775129; doi:10.3389/fpls.2013.00369)
Supplement: Supplementary file 2 [file DataSheet2.DOCX]

**Supplemental Material 2**

Boxplots of all 109 metabolites. Normalized relative peak area for each metabolite is plotted in arbitrary units for each factor combination (organs, genotypes and S-fertilization rate). Wild type, wt; transgene, tg; 0 g S/pot, S0; 0.1 g S/pot, S1; 0.2 g S/pot, S1.


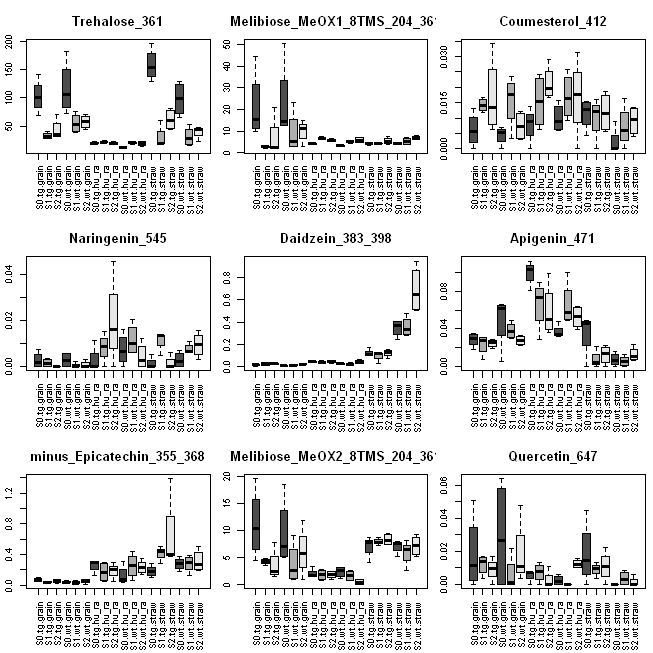


Supplemental Material 2 (*continued*)


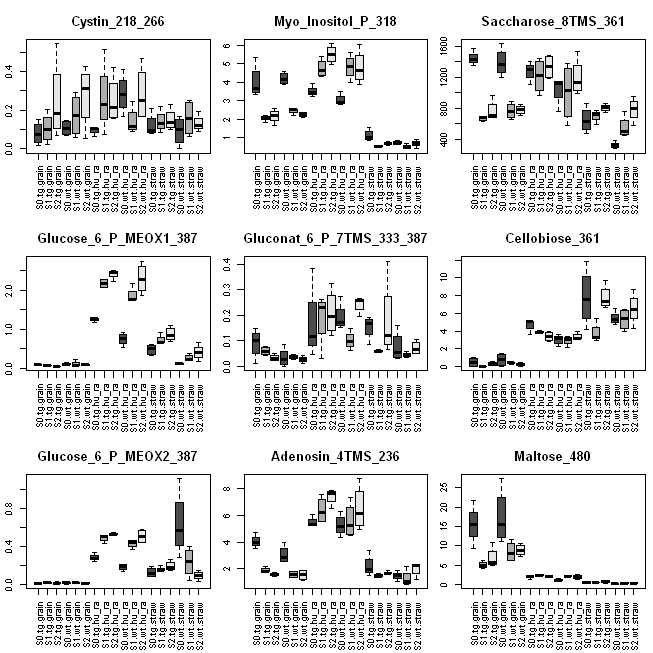


Supplemental Material 2 (*continued*)


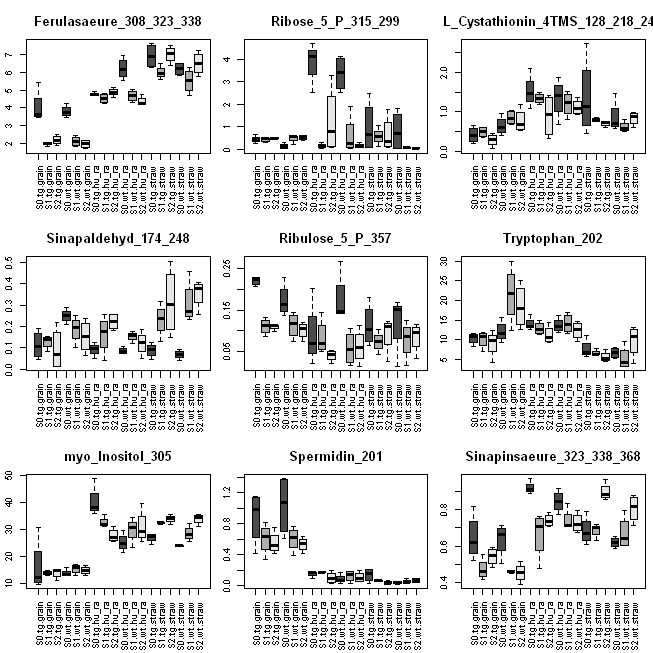


Supplemental Material 2 (*continued*)


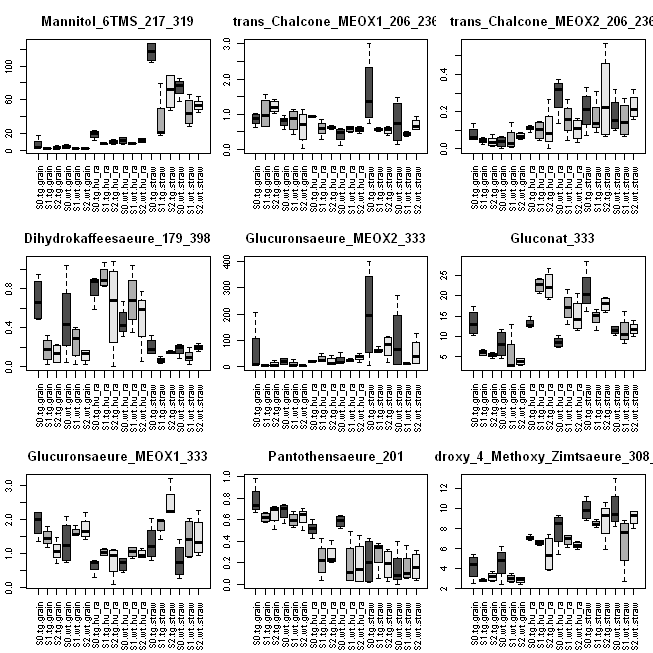


Supplemental Material 2 (*continued*)


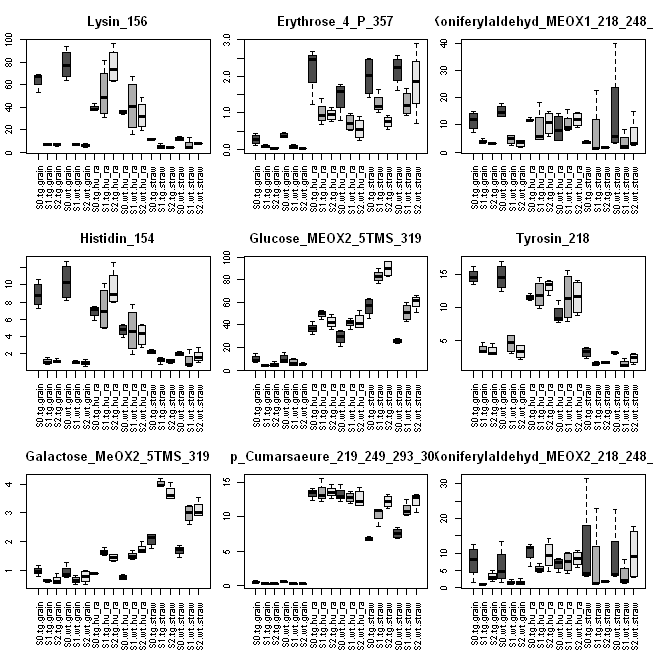


Supplemental Material 2 (*continued*)


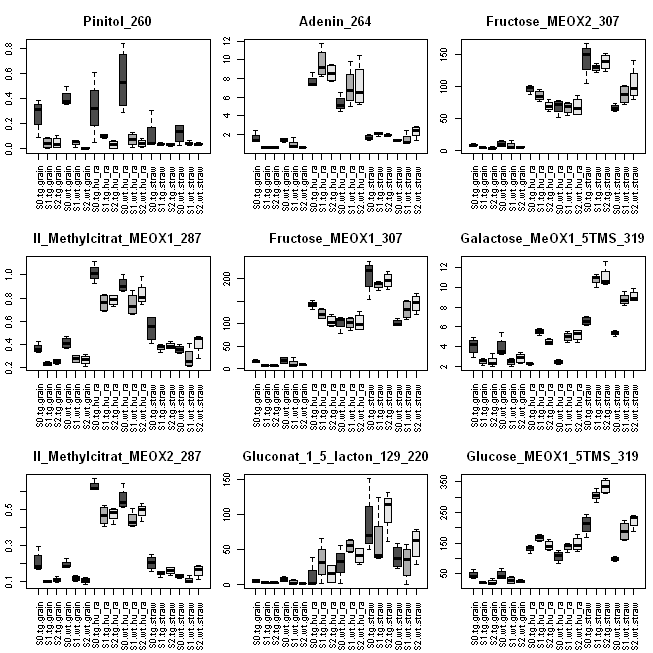


Supplemental Material 2 (*continued*)


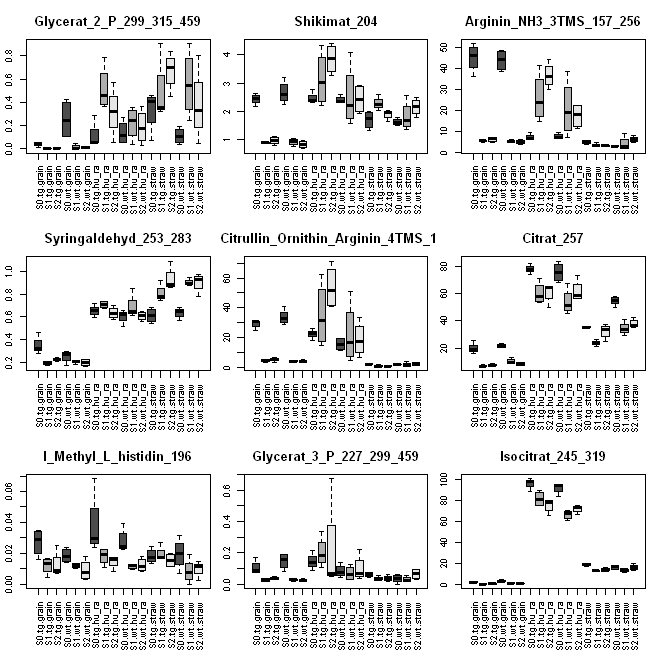


Supplemental Material 2 (*continued*)


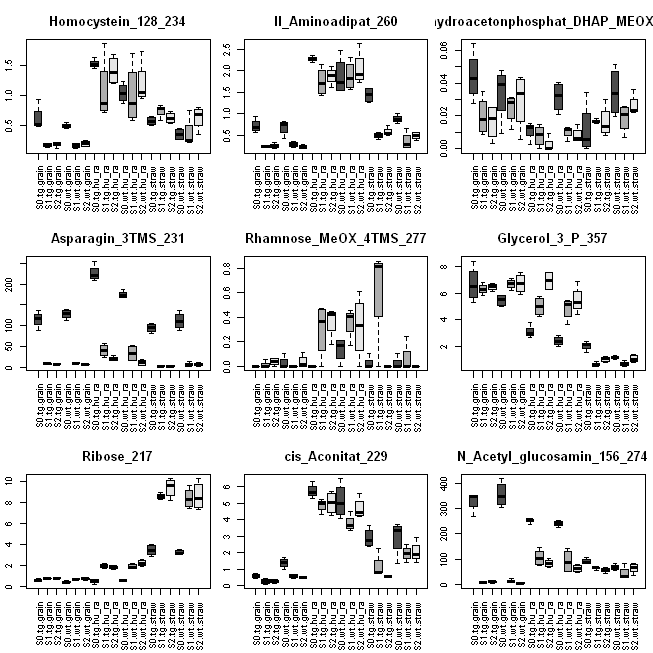


Supplemental Material 2 (*continued*)


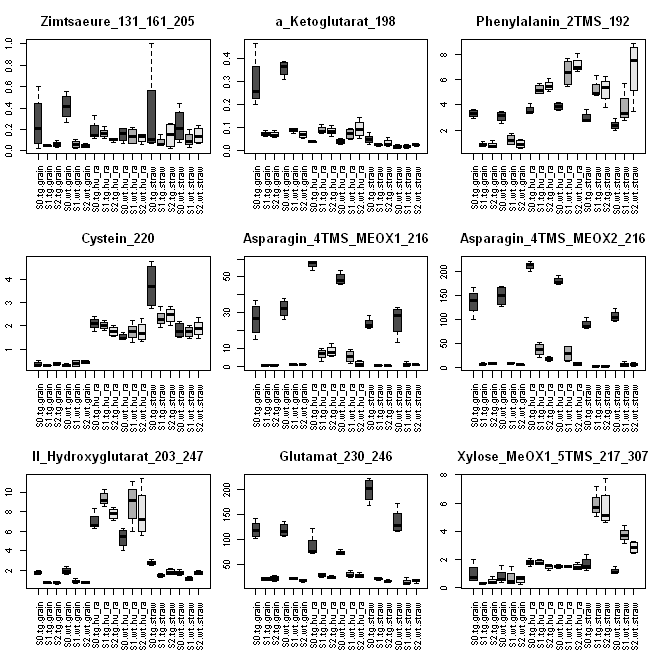


Supplemental Material 2 (*continued*)


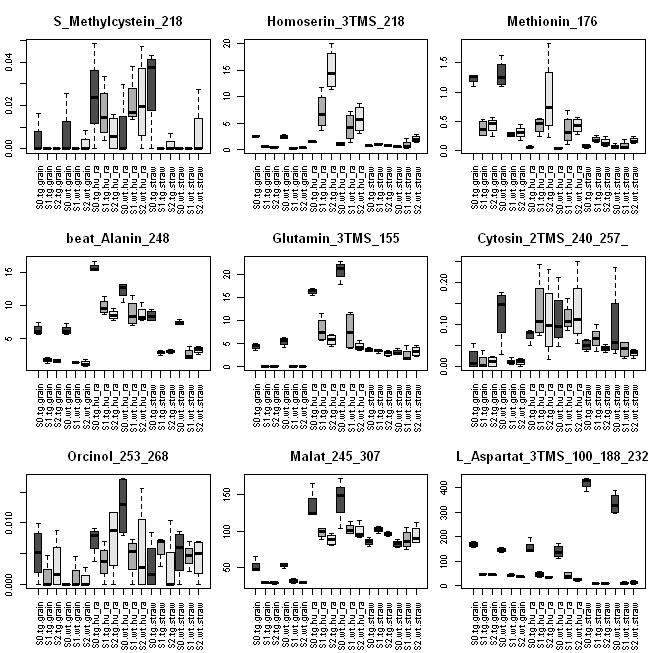


Supplemental Material 2 (*continued*)


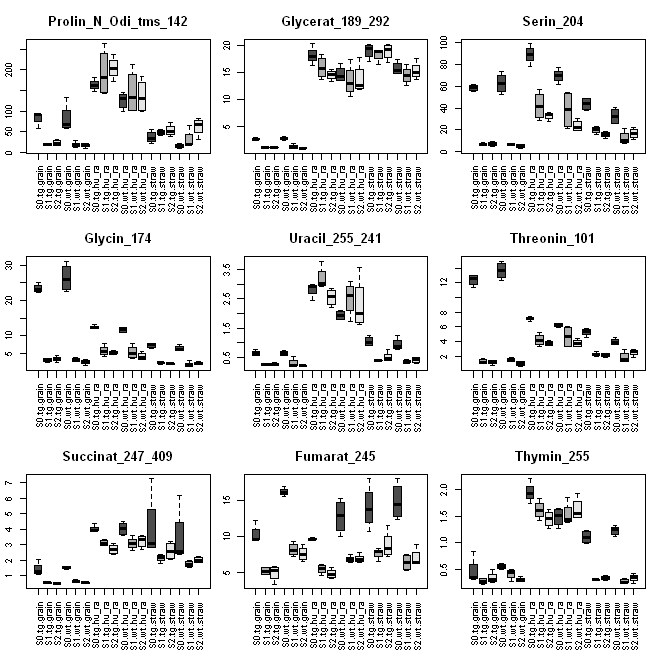


Supplemental Material 2 (*continued*)


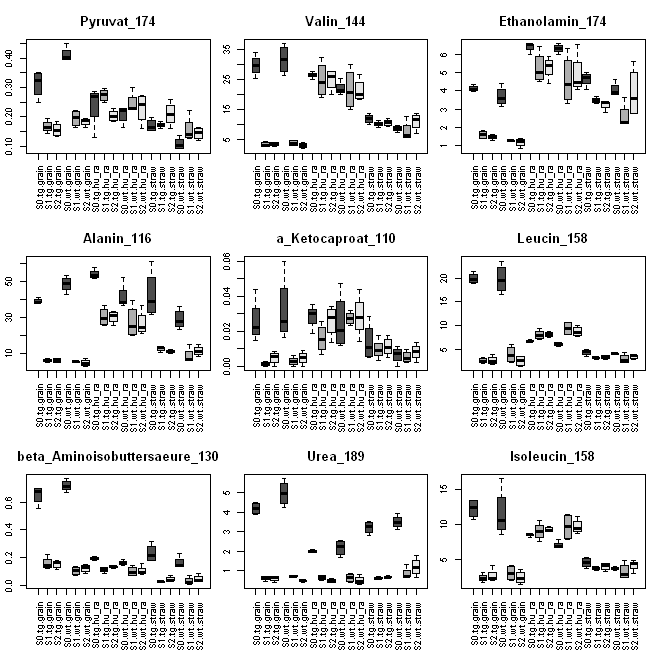


Supplemental Material 2 (*continued*)


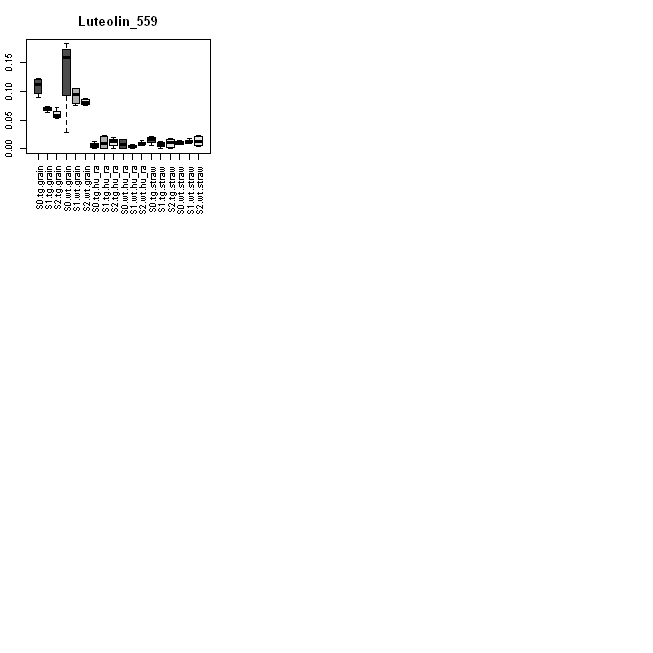


Supplemental Material 2 (*continued*)
