# Supplementary material for: Silencing of the sulfur rich α-gliadin storage protein family in wheat grains (Triticum aestivum L.) causes no unintended side-effects on other metabolites
Source: Front Plant Sci. 2013 Sep 17;4:369. doi: 10.3389/fpls.2013.00369 (PMC3775129; doi:10.3389/fpls.2013.00369)

**Supplemental Material**

**Supplemental Material 3**

*Left graph*: Screeplot of the PCA. The first three principal components have proportions of variance of 0.3018, 0.2339 and 0.1185. Since the first principal component explains, at 30.18%, most of the variances on the PCA, subsequent multiple contrast tests for ratios of means (compare tables 1 and 2) were based on the first principal components*. Right graph:* Principal component analysis (PCA) of metabolite data obtained from the two genotypes. Loading plot (related to the first and second principal component) for all metabolites.


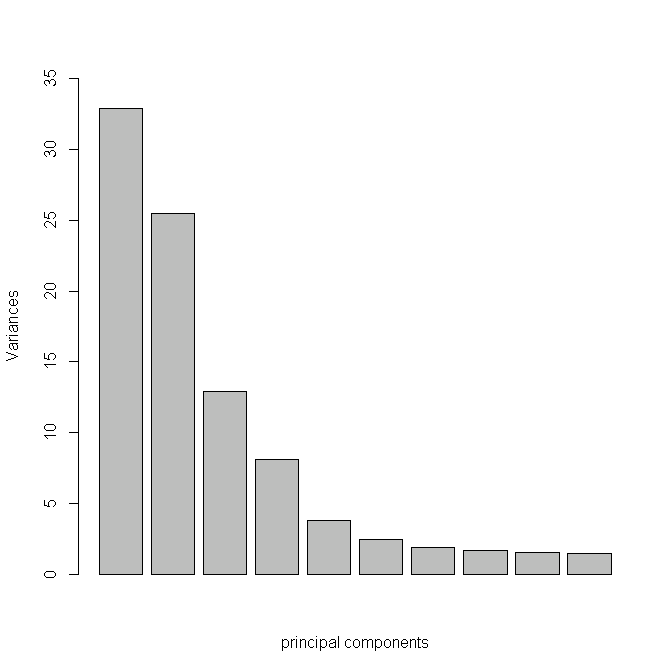

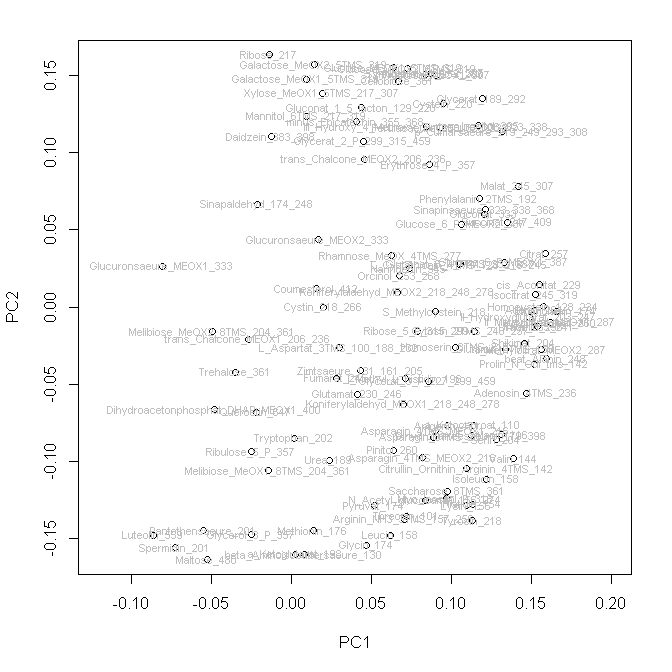

Supplement: Supplementary file 3 [file DataSheet3.DOCX]
