# Supplementary material for: Silencing of the sulfur rich α-gliadin storage protein family in wheat grains (Triticum aestivum L.) causes no unintended side-effects on other metabolites
Source: Front Plant Sci. 2013 Sep 17;4:369. doi: 10.3389/fpls.2013.00369 (PMC3775129; doi:10.3389/fpls.2013.00369)

**Supplemental Material 4**

Principal component analysis (PCA) of metabolite data obtained from the two genotypes. Loading plot (related to the first and second principal component) for the 28 most important metabolites.


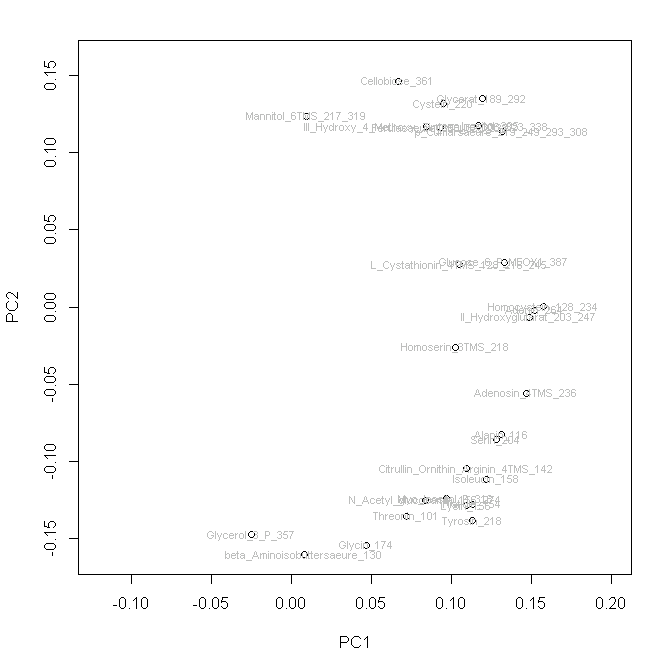

Supplement: Supplementary file 4 [file DataSheet4.DOCX]
